# Supplementary material for: Transcriptome analysis of shade-induced growth and photosynthetic responses in soybean cultivars
Source: PLoS One. 2025 Sep 10;20(9):e0332271. doi: 10.1371/journal.pone.0332271 (PMC12422440; doi:10.1371/journal.pone.0332271)
Supplement: Attachment — Submitted filename: point-by-point response report.docx [file pone.0332271.s005.docx]

**Response to Editor and Reviewers**

We thank the Academic Editor and both reviewers for their constructive comments and suggestions. We have carefully addressed each point raised and believe the manuscript has been significantly improved. Below is our detailed point-by-point response.

**EDITORIAL REQUIREMENTS**

**Editor Comment: Please ensure that your manuscript meets PLOS ONE's style requirements, including those for file naming.**

**Response**: We have reformatted the manuscript according to PLOS ONE style requirements using the provided templates. All file naming conventions have been followed as specified in the guidelines.

**Editor Comment: We noticed minor occurrence of overlapping text with previous publications that needs to be addressed.**

**Response**: We have identified and addressed all instances of overlapping text with the cited publications.

Editor Comment: Please provide an amended funding statement and remove funding information from Acknowledgments.

**Response**: We have amended our Funding Statement as follows: This work was supported by Natural Science Foundation of Heilongjiang Province Project (LH2023C095); The 2024 Science and Technology Support Project of the Inner Mongolia Innovation Center of Biological Breeding Technology -Biotechnology-Based Breeding of High-Quality Soybeans and Application Demonstration (2024NSZC04).

All funding-related texts have been removed from the Acknowledgments section.

**Acknowledgments:** Special thanks to the Soybean intellect design breeding laboratory of Heilongjiang Academy of Agricultural Sciences for providing platform support. Soybean Germplasm Resources Team of the Institute of Crop Sciences, Chinese Academy of Agricultural Sciences for providing soybean germplasm resources.

**Editor Comment: Please include direct links to access each database.**

**Response**: We have updated our Data Availability Statement to include: "Raw sequencing data are available in the NCBI Sequence Read Archive (SRA) under BioProject accession number PRJNA1210143 (https://www.ncbi.nlm.nih.gov/bioproject/?term=PRJNA1210143).

**Editor Comment: Please include captions for Supporting Information files at the end of your manuscript.**

**Response**: We have added comprehensive captions for all Supporting Information files at the end of the manuscript:

- Table S1: RNA-sequencing libraries quality metrics including read counts, GC content, and mapping statistics for all samples
- Table S2: Detailed quality control measures for RNA libraries, including filtration parameters and alignment percentages

**REVIEWER #1 COMMENTS**

Major Comments

1. The P-Value used is 0.01 while no information about FDR. At least the p-value should be
0.05, which is a good statistical significance for DEGs. It is essential to apply multiple
testing correction, such as the Benjamini-Hochberg procedure, to control the false
discovery rate (FDR) during multiple comparisons.

**Response**: We agree with you that these points are critical. We have revised our statistical analysis approach, ensuring the following updates:

1- We changed P-value threshold changed from p < 0.01 to adjusted p-value (FDR) < 0.05. We also made an FDR correction as we implemented the Benjamini-Hochberg procedure to control the false discovery rate
2- In the Methods, an update was made via dding detailed description in the RNA-Seq data analysis section, as follows:

"*Raw p-values were adjusted using the Benjamini-Hochberg procedure to control the false discovery rate (FDR). Genes were classified as differentially expressed if they satisfied the following criteria: adjusted p-value (FDR) < 0.05, |log₂ fold change| > 1, and mean normalized count > 10 across treatment groups*."

**2. The specific method used for DEG analysis is not mentioned.**

**Response**: We have added comprehensive details about the DEG analysis methodology, as follows:
"*Differential gene expression analysis was conducted using DESeq2 (Version 1.34.0) in R (Version 4.1.3). Count matrices were imported and genes with mean counts < 10 across all samples were filtered. Size factors were calculated using DESeq2's median-of-ratios method. Gene-wise dispersion parameters were estimated using the parametric fit method with local regression and empirical Bayes shrinkage. Differential expression was tested using the Wald test implemented in DESeq2, which models count data using a negative binomial generalized linear model*."

**Minor Comments**

**1. Too much general background, much repeated in later sections. Shorten and focus on research gap and novelty (Lines 39–80).**

**Response**: We have condensed the Introduction by reducing background information on soybean, removing redundant content from the discussion, and highlighting the novelty of the study: a comparative transcriptomic analysis across multiple shade levels with contrasting genotypes.

**2. Inconsistent use of cultivar names throughout (Lines 20, 109, 200): "Guru" and "GURU" are used interchangeably. Use consistent case and formatting for cultivar names.**

**Response**: We have standardized all cultivar names throughout the manuscript.

**3. The rationale is clear, but a formal hypothesis is not stated. Add a hypothesis or explicit
research objective at the end of the introduction (Lines 81–88).**

**Response**: We have added a clear hypothesis and objectives: "*We hypothesized that shade-tolerant and shade-sensitive cultivars would show distinct transcriptomic profiles under shade stress, with the tolerant cultivar exhibiting enhanced genes related to light capture and photosynthesis, while the sensitive cultivar shows shade avoidance responses. This study aims to: (1) characterize physiological and morphological responses of contrasting genotypes to graded shade stress, (2) identify differentially expressed genes and pathways linked to shade tolerance, and (3) correlate key transcriptomic signatures with shade tolerance traits*."

**4. Replicate numbers are not fully defined for RNA-seq experiments. Include biological
replicate numbers per treatment and confirm sequencing depth per sample (Lines
119–120, 209–215).**

**Response**: We have clarified the experimental design: "Each treatment included three biological replicates with six plants per replicate (n = 18 plants per treatment combination). For RNA-seq analysis, leaf samples were collected from three biological replicates per treatment, with each replicate representing pooled material from two plants to ensure adequate RNA yield while maintaining biological independence."

**5. Lists of DEGs and comparisons (e.g., G70 vs. H70, GCK vs. HCK) are provided without
sufficient biological insight. Focus on top representative genes and discuss their
functional roles more deeply (Lines 217–238).**

**Response**: We have enhanced the biological interpretation, adding the following updates:

- Adding detailed functional analysis of key DEG categories
- Discussing specific gene families and their roles (e.g., calcium-dependent protein kinases, histone deacetylases)
- Providing mechanistic explanations for how these genes contribute to shade tolerance

**6. “Net photosynthetic rate (pr)” should be “(Pn)” for standardization. Correct abbreviation to avoid misinterpretation (Line 180).**

**Response**: Corrected throughout the manuscript. All instances now use the standard abbreviation "(Pn)" for net photosynthetic rate.

**7. “One highly shade-tolerant” should be “One cultivar, Guru, which is highly shade-tolerant-
tolerant” (Line 92), “do more photosynthesis” should be “perform more photosynthesis”
(Line 195).**

**Response**: Thanks for valuable comment, we rephrased these parts based on your comment.

**8. Redundancy in the Discussion with Lines 296–338 and repeated in 334–344. Repetition
in describing chlorophyll content, photosynthesis, and light adaptation mechanisms.
Consolidate overlapping parts.**

**Response**: Thanks again, we have completely restructured the Discussion section to eliminate redundancy:

**9. Incorrect figure reference in text. “Figure 3C” mentioned in the context of DEG
comparisons likely should be “Figure 4c”. Verify all figure citations match figure labels.**

**Response**: Corrected all figure references. The text now correctly references "Figure 4c" for DEG comparisons. All figure citations have been verified to match figure labels.

**10. Improper listing (repeated GO terms without separators, e.g., “GO:0003712),
(GO:0004683)”). Ensure proper punctuation in gene ontology listings (Lines 259–260).**

**Response**: All GO term listings have been reformatted with proper punctuation and separators for clarity and readability.

**11. Consistency in writing numbers throughout the manuscript. Mix of numerical and word formats (e.g., “three treatments” vs. “3 shade treatments”). Use consistent numerical
formatting.**

**Response**: We have standardized number formatting according to scientific writing conventions:

**REVIEWER #2 COMMENTS**

Reviewer #2: The manuscript entitled “Transcriptome Analysis of Shade-Induced Growth and Photosynthetic Responses in Soybean Cultivars” presents a well-designed and comprehensive study investigating the physiological, morphological, and transcriptomic responses of two contrasting soybean cultivars—Guru (shade-tolerant) and Heinong 53 (shade-sensitive)—under varying levels of shade (0%, 30%, and 70%). Through a combination of phenotypic analysis, chlorophyll and photosynthesis measurements, microscopic evaluation, and RNA sequencing, the authors demonstrate that Guru maintains higher photosynthetic efficiency, chlorophyll content, and a more stable resource allocation strategy under shade stress compared to Heinong 53, which exhibits excessive stem elongation and photosynthetic decline. The transcriptome analysis identified 2,596 differentially expressed genes (DEGs), with enriched functions related to shade avoidance, photosynthesis, and stress responses, including histone deacetylase and calcium-dependent kinase activity. Strong correlations between specific DEGs and physiological traits such as net photosynthetic rate and plant height were observed, supporting the molecular basis of phenotypic differences. This integrative work advances our understanding of shade tolerance mechanisms and provides valuable targets for breeding more resilient soybean cultivars. The manuscript is well-structured, methodologically sound, and the data are convincingly presented; I have no objections to its acceptance in its current form.

**Response**: We thank Reviewer 2 for the positive evaluation.
