# Supplementary material for: Transcriptome analysis of shade-induced growth and photosynthetic responses in soybean cultivars
Source: PLoS One. 2025 Sep 10;20(9):e0332271. doi: 10.1371/journal.pone.0332271 (PMC12422440; doi:10.1371/journal.pone.0332271)
Supplement: Attachment — Submitted filename: Reviewer comments.docx [file pone.0332271.s004.docx]

**Reviewer #1:** The manuscript titled "Transcriptome Analysis of Shade-Induced Growth and
Photosynthetic Responses in Soybean Cultivars" is a comprehensive multi-level analysis
(morphological, physiological, transcriptomic) with strong data interpretation supported by
statistical testing. The manuscript have good potential for real-world application in breeding
shade-tolerant soybean cultivars. Following are the some major/minor corrections suggested
before the acceptance of the manuscript:
Major:
1. The P-Value used is 0.01 while no information about FDR. At least the p-value should be
0.05, which is a good statistical significance for DEGs. It is essential to apply multiple
testing correction, such as the Benjamini-Hochberg procedure, to control the false
discovery rate (FDR) during multiple comparisons.
2. The specific method used for DEG analysis is not mentioned.
Minor:
1. Too much general background on soybean and light effects, much of which is repeated in
later sections. Shorten and focus more on the research gap and novelty of this study
(Lines 39–80).
2. Inconsistent use of cultivar names throughout (Lines 20, 109, 200): "Guru" and "GURU"
are used interchangeably. Use consistent case and formatting for cultivar names.
3. The rationale is clear, but a formal hypothesis is not stated. Add a hypothesis or explicit
research objective at the end of the introduction (Lines 81–88).
4. Replicate numbers are not fully defined for RNA-seq experiments. Include biological
replicate numbers per treatment and confirm sequencing depth per sample (Lines
119–120, 209–215).
5. Lists of DEGs and comparisons (e.g., G70 vs. H70, GCK vs. HCK) are provided without
sufficient biological insight. Focus on top representative genes and discuss their
functional roles more deeply (Lines 217–238).
6. “Net photosynthetic rate (pr)” should be “(Pn)” for standardization. Correct abbreviation
to avoid misinterpretation (Line 180).

7. “One highly shade-tolerant” should be “One cultivar, Guru, which is highly shade-
tolerant” (Line 92), “do more photosynthesis” should be “perform more photosynthesis”
(Line 195).
8. Redundancy in the Discussion with Lines 296–338 and repeated in 334–344. Repetition
in describing chlorophyll content, photosynthesis, and light adaptation mechanisms.
Consolidate overlapping parts.
9. Incorrect figure reference in text. “Figure 3C” mentioned in the context of DEG
comparisons likely should be “Figure 4c”. Verify all figure citations match figure labels.
10. Improper listing (repeated GO terms without separators, e.g., “GO:0003712),
(GO:0004683)”). Ensure proper punctuation in gene ontology listings (Lines 259–260).
11. Consistency in writing numbers throughout the manuscript. Mix of numerical and word
formats (e.g., “three treatments” vs. “3 shade treatments”). Use consistent numerical
formatting.

**Reviewer #2:** The manuscript entitled “Transcriptome Analysis of Shade-Induced Growth and Photosynthetic Responses in Soybean Cultivars” presents a well-designed and comprehensive study investigating the physiological, morphological, and transcriptomic responses of two contrasting soybean cultivars—Guru (shade-tolerant) and Heinong 53 (shade-sensitive)—under varying levels of shade (0%, 30%, and 70%). Through a combination of phenotypic analysis, chlorophyll and photosynthesis measurements, microscopic evaluation, and RNA sequencing, the authors demonstrate that Guru maintains higher photosynthetic efficiency, chlorophyll content, and a more stable resource allocation strategy under shade stress compared to Heinong 53, which exhibits excessive stem elongation and photosynthetic decline. The transcriptome analysis identified 2,596 differentially expressed genes (DEGs), with enriched functions related to shade avoidance, photosynthesis, and stress responses, including histone deacetylase and calcium-dependent kinase activity. Strong correlations between specific DEGs and physiological traits such as net photosynthetic rate and plant height were observed, supporting the molecular basis of phenotypic differences. This integrative work advances our understanding of shade tolerance mechanisms and provides valuable targets for breeding more resilient soybean cultivars. The manuscript is well-structured, methodologically sound, and the data are convincingly presented; I have no objections to its acceptance in its current form.
